# Clinical validation of a novel quantitative assay for the detection of *MGMT* methylation in glioblastoma patients

**DOI:** 10.1186/s13148-021-01044-2

**Published:** 2021-03-09

**Authors:** Rocio Rosas-Alonso, Julian Colmenarejo-Fernandez, Olga Pernia, Carlos Rodriguez-Antolín, Isabel Esteban, Ismael Ghanem, Dario Sanchez-Cabrero, Itsaso Losantos-Garcia, Sara Palacios-Zambrano, Gema Moreno-Bueno, Javier de Castro, Virginia Martinez-Marin, Inmaculada Ibanez-de-Caceres

**Affiliations:** 1 Epigenetics Laboratory. INGEMM, Paseo La Castellana 261. Edificio Bloque Quirúrgico Planta -2. University Hospital La Paz, 28046 Madrid, Spain; 2 Experimental Therapies and Novel Biomarkers in Cancer. IdiPAZ, Madrid, Spain; 3 Pathology Department, La Paz University Hospital, Madrid, Spain; 4 Medical Oncology Department, La Paz University Hospital, Madrid, Spain; 5 Biostatistics Unit, La Paz University Hospital. IdiPAZ, Madrid, Spain; 6 MD Anderson Cancer Center, Madrid, Spain; 7 Biochemistry Department, UAM/ IIBm (CSIC-UAM), IdiPaz, Fundación MD Anderson Internacional, Madrid, Spain; 8 CIBERONC, Madrid, Spain

**Keywords:** *MGMT* methylation, MSP, Dp_qMSP, Glioblastoma

## Abstract

**Background:**

The promoter hypermethylation of the methylguanine-DNA methyltransferase gene is a frequently used biomarker in daily clinical practice as it is associated with a favorable prognosis in glioblastoma patients treated with temozolamide. Due to the absence of adequately standardized techniques, international harmonization of the *MGMT* methylation biomarker is still an unmet clinical need for the diagnosis and treatment of glioblastoma patients.

**Results:**

In this study we carried out a clinical validation of a quantitative assay for MGMT methylation detection by comparing a novel quantitative MSP using double-probe (dp_qMSP) with the conventional MSP in 100 FFPE glioblastoma samples. We performed both technologies and established the best cutoff for the identification of positive-methylated samples using the quantitative data obtained from dp_qMSP. Kaplan–Meier curves and ROC time dependent curves were employed for the comparison of both methodologies.

**Conclusions:**

We obtained similar results using both assays in the same cohort of patients, in terms of progression free survival and overall survival according to Kaplan–Meier curves. In addition, the results of ROC(t) curves showed that dp_qMSP increases the area under curve time-dependent in comparison with MSP for predicting progression free survival and overall survival over time. We concluded that dp_qMSP is an alternative methodology compatible with the results obtained with the conventional MSP. Our assay will improve the therapeutic management of glioblastoma patients, being a more sensitive and competitive alternative methodology that ensures the standardization of the *MGMT*-biomarker making it reliable and suitable for clinical use.

**Supplementary Information:**

The online version contains supplementary material available at 10.1186/s13148-021-01044-2.

## Background

Epigenetic modifications are a hallmark of human cancers. The reduction of tumor-associated methylation levels which is associated with genomic instability was one of the first epigenetic alterations to be described [1]. However, there are some areas of the genome that increase their methylation levels, which normally correspond with CpG islands of tumor suppressor genes [2–4]. DNA methylation is catalyzed by DNA methyl-transferases, which transfer methyl groups from S-adenosylmethionine on CpG dinucleotides at the 5′carbon position of cytosines located at CpG islands. Methyl groups are recognized by Methyl-CpG-binding domain proteins, which interfere with the binding of transcriptional activators of DNA [5].

The methylguanine-DNA methyltransferase (*MGMT*) gene promoter hypermethylation is one of the most studied molecular biomarkers in neuro-oncology. *MGMT* gene encodes a repair enzyme that removes alkyl groups from the O^6^ position of guanine and works by antagonizing the cytotoxic effects of alkylating agents [6]. Promoter methylation is the main way of silencing the *MGMT* gene and predicts a favorable outcome in glioblastoma patients treated with alkylating drugs. Glioblastoma (GBM) is the most common primary malignant central nervous system tumor in adults and is invariably associated with poor prognosis. Only 33% of patients survive one year and only 5% of patients live more than five years after diagnosis [7–9]. Thus, the methylation status of *MGMT* is frequently used in the daily clinical routine as a predictive biomarker to classify GBM patients who are more likely to respond to temozolamide.

The *MGMT* CpG island has 98 CpG sites located on chromosome 10q26 that controls the *MGMT* gene expression. Malley et al*.* defined a differentially methylated region (DMR2) essential for silencing the *MGMT* gene. Most of the assays are based on the analysis of the CpG sites 73 to 90 located at the DMR2 area. Throughout this area, the CpGs 83, 86, 87 and 89 have been the best targets for methylation testing [10]. Furthermore, Bady et al*.* described two CpG sites in the *MGMT* promoter (cg12434587, chr10:131,265,209–131,265,210 and cg12981137, chr10:131,265,575–131,265,576) that showed the strongest association with overall survival (OS), being cg12981137 the CpG number 84 in the DMR2 area, and supporting the idea proposed by Malley et al*.* [11].

A wide range of molecular assays are available for qualitative and quantitative *MGMT* methylation detection. The most commonly used methods are based on bisulfite conversion of unmethylated cytosines into uracil [12]. Examples of methods include methylation-specific PCR (MSP) [6, 13, 14], pyrosequencing [13–15], different variations of real-time PCR [14, 16], digital PCR [17], methylation-specific multiplex ligation-dependent probe amplification (MS-MLPA) [13, 18], methylation-specific high-resolution melting (HRM) [19], and combined bisulfite restriction analysis (COBRA) [20]. Other techniques that can evaluate global methylation changes such as next-generation sequencing are currently employed in the field of research but not in the routine clinical practice [21].Currently, MSP and pyrosequencing are the most widely used technical approaches to *MGMT* methylation analysis, providing information that is useful for clinical decision-making. However, the analytical sensitivity differs considerably among diverse assays and their standardization across a wide range of diagnostic laboratories is lacking [22, 23]. In fact, there is still a lack of consensus on how to interpret the pyrosequencing data [14, 15]. In addition to the method used, other factors such as tumor content, contamination of inflammatory and stromal cells, necrosis, and tumor heterogeneity could affect the methylation results obtained [24].

Due to the increasing interest in molecular biomarkers and their impact in therapeutic management of glioblastoma patients, more sensitive and competitive alternative methodologies are in demand. In this study, we have developed an innovative quantitative methylation specific PCR (dp_qMSP) assay used for the study of *MGMT* methylation and validated its clinical use by comparing this novel assay with the conventional MSP.

## Results

### Clinical data

From May 2014 to March 2020, we enrolled 100 patients with newly diagnosed GBM. Among the 100 patients, 42 were women and 58 were men. The average age at diagnosis was 61 years old (age range 24–83 years).

No significant differences were found between patients’ age, sex, type of surgery, ECOG and *MGMT* promoter methylation assessed with MSP or dp_qMSP. Relevant clinical data of patients are described in Table 1.

**Table 1:** Demographic and clinical data of the study population (*n* = 100)

Characteristic	Value	Methylation status MSP	Methylation status dp_qMSP	MSP (*p *value)	dp_qMSP (*p* value)
Average age at surgery and range	61 (25—84)			*p* = 0.632	*p* = 0.697
Sex					
Women	42	15 methylated 27 unmethylated	19 methylated 23 unmethylated	*p* = 0.377	*p* = 0.218
Men	58	15 methylated 43 unmethylated	19 methylated 39 unmethylated		
Type of surgery					
Total resection	51	19 methylated 32 unmethylated	22 methylated 29 unmethylated	*p* = 0.174	*p* = 0.367
Partial resection	29	8 methylated 21 unmethylated	11 methylated 18 unmethylated		
Biopsy	20	3 methylated 17 unmethylated	5 methylated 15 unmethylated		
ECOG					
0	59	17 methylated 42 unmethylated	21 methylated 38 unmethylated	*p* = 0.727	*p* = 0.624
1	24	7 methylated 17 unmethylated	10 methylated 14 unmethylated		
2	12	5 methylated 7 unmethylated	6 methylated 6 unmethylated		
3	5	1 methylated 4 unmethylated	1 methylated 4 unmethylated		

### Comparison between dp_qMSP and MSP methods for MGMT promoter methylation detection

**Fig. 1:**
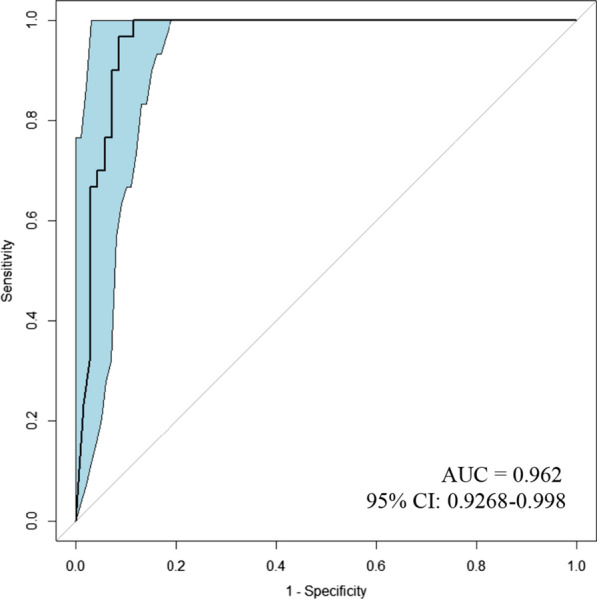
ROC curve for dp_qMSP compared to MSP (*n* = 100). Area under the curve = 0.962 (95% CI 0.9268–0.998). Blue shade represents the CI

**Fig. 2:**
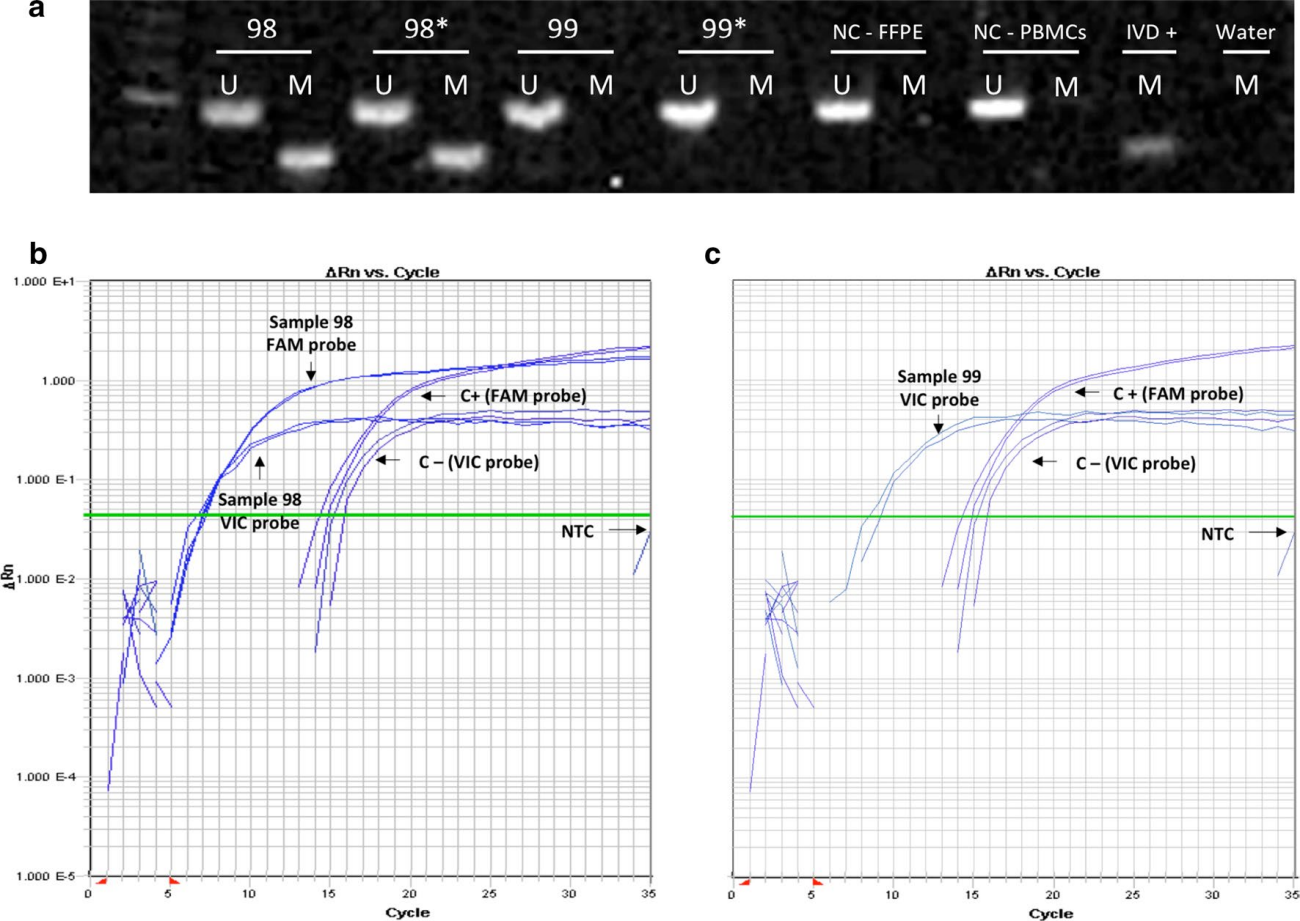
MSP and dp_qMSP examples in the analyzed tumor samples for patients 98 and 99. **a** Example of *MGMT* promoter methylation in acrylamide gel. The MSP products were loaded and electrophoresed as follows: sample number 98 (lanes 1–4 unmethylated and methylated reactions performed by duplicated), sample number 99 (lanes 5–8, unmethylated and methylated reactions performed by duplicated), lanes 9–10 correspond to unmethylated and methylated reactions using a FFPE negative control sample. Lanes 11 and 12 correspond to unmethylated and methylated reactions using a PBMC control sample. Lane 13 corresponds to PBMC methylated in vitro (IVD) as a positive control, and last line is the water methylation reaction used to discard contamination. **b, c**. Example of methylated and unmethylated amplification by qMSP. B. Patient number 98, FAM (M) and VIC (U) probes amplified (47.9% methylation). **c** Patient number 99, only VIC probe amplified (0% methylation). U: Unmethylated. M: methylated. FFPE: Formalin fixed paraffin embedded. PBMCs: Peripheral blood mononuclear cells. NC: negative control. IVD: In vitro Methylated DNA (positive control). NTC (No Template Control)

ROC curve was performed to determine the cutoff for dp_qMSP. The area under the curve (AUC) was 0.962 (95% CI 0.927–0.998) (Fig. 1). The methylation cutoff point was established in 3.75% and was obtained by the formula previously described [25]. Thus, the samples were classified as methylated when the methylation was above the cutoff point of 3.75% and unmethylated when they were less than 3.75%. The sensitivity and specificity for this cutoff point were 100% (95% CI 88.6–100) and 88.6% (95% CI 79.0–94.1), respectively. *MGMT* methylation was detected in 30 out of 100 FFPE samples by MSP and 38 out of 100 samples by dp_qMSP (Table 2); a representative gel and quantitative amplifications are shown in Fig. 2 (see the uncropped gel at Additional file 1: Fig. 1). We obtained discrepancies in eight samples within both technologies, two of these eight patients present a survival in the mean value of patients harboring a methylated promoter (> 18 months) (Patients number 1; 23.4 months and patient number 76; 21.6 months). Patients number 75 and 100 were alive at their last following-up at our hospital, although unfortunately we lost their follow-up because they changed hospitals. Patients number 28 and 78, with median survival of 8.3 months with a incomplete tumor resection and 12,6 months with complete resection, respectively present a standard overall survival in this pathology and the last two patients (numbers 89; 4.5 months and 91; 4.8 months) with the worst survival, were diagnosed with biopsy and they did not underwent a complete resection. The results from those samples by MSP and dp_qMSP together with sample number 11 that presents the lowest percentage of methylation by using dp_qMSP are shown in Additional file 2: Supplementary Fig. 2. If we consider the eight positives identified by dp_qMSP to be false positives based on the data obtained using MSP, the specificity achieved by dp_qMSP would be 88.6%. Additionally, we considered of great interest to probe the presence of methylated DNA molecules in these samples, and in fact, in collaboration with the Md Anderson hospital, they were able to amplify 5 of these samples using an alternative MSP technique with different settings and DNA modification procedures (deeply described in Additional file 3: supplementary Fig. 3) and found a very weak amplification at the methylated reaction in three of the samples, 1, 78 and 100. In none of the three cases, this amplification would have suggested the diagnosis of a methylated sample for the *MGMT* marker, as it happens with our results using MSP technology, but supports our positive results obtained by dp_qMSP, as these three samples out of the five, are the ones with the highest percentage of methylation when were analysed by dp_qMSP in our laboratory.

**Table 2:** Clinical, pathological and methylation data of 100 GBM patients

ID	Age	Sex	Type of surgery	ECOG	MSP	%Methylation dp_qMSP
1	57	Female	Total resection	0	U	66,6
2	70	Male	Biopsy	2	U	0
3	45	Male	Partial resection	0	M	65
4	44	Male	Partial resection	0	U	0
5	54	Male	Partial resection	0	U	0
6	71	Male	Total resection	0	U	1,6
7	61	Female	Total resection	0	M	60,3
8	47	Female	Total resection	0	M	66
9	77	Female	Biopsy	3	U	0
10	49	Male	Biopsy	0	U	0
11	69	Female	Partial resection	2	M	5,9
12	67	Male	Total resection	0	U	0
13	65	Female	Total resection	0	U	0
14	80	Female	Total resection	0	U	0
15	66	Female	Partial resection	0	U	0
16	65	Female	Biopsy	1	U	0
17	81	Female	Biopsy	0	U	0
18	72	Male	Partial resection	0	M	97,5
19	54	Male	Partial resection	3	U	0
20	51	Male	Total resection	0	U	0
21	49	Male	Total resection	3	U	0
22	79	Female	Total resection	2	M	99,9
23	55	Female	Partial resection	1	U	0
24	64	Male	Total resection	0	M	27,5
25	49	Male	Total resection	0	M	94,1
26	62	Female	Total resection	0	M	97,6
27	55	Male	Partial resection	0	U	0
28	65	Male	Partial resection	0	U	64,6
29	58	Male	Partial resection	0	U	0
30	71	Female	Total resection	0	U	0
31	76	Female	Total resection	0	M	17,8
32	73	Female	Total resection	0	M	83
33	67	Male	Total resection	0	U	0
34	60	Male	Total resection	0	U	0
35	50	Female	Total resection	0	M	36
36	54	Male	Total resection	0	M	96,4
37	62	Male	Biopsy	0	U	0
38	76	Female	Biopsy	0	M	100
39	76	Female	Total resection	1	U	0
40	84	Male	Biopsy	0	U	0
41	75	Male	Total resection	1	M	100
42	46	Male	Total resection	0	U	0
43	55	Female	Partial resection	2	U	0
44	80	Female	Partial resection	1	U	0
45	29	Male	Biopsy	0	M	77
46	56	Male	Biopsy	1	U	0
47	61	Male	Total resection	1	U	0
48	72	Male	Total resection	0	M	92,6
49	48	Female	Partial resection	1	M	100
50	66	Male	Total resection	0	U	0
51	70	Male	Partial resection	0	U	0
52	82	Male	Biopsy	3	M	99,9
53	52	Female	Total resection	0	U	0
54	54	Female	Total resection	0	M	100
55	68	Female	Biopsy	2	U	0
56	60	Male	Total resection	0	U	0
57	54	Male	Partial resection	0	U	0
58	64	Male	Partial resection	0	U	0
59	75	Female	Total resection	0	M	88,89
60	68	Female	Partial resection	1	U	0
61	73	Female	Partial resection	1	U	0
62	51	Male	Total resection	1	U	0
63	37	Male	Total resection	1	M	100
64	69	Male	Biopsy	0	U	0
65	71	Male	Total resection	0	M	100
66	67	Female	Total resection	1	U	0
67	51	Male	Total resection	2	M	99,9
68	50	Male	Total resection	0	U	0
69	57	Female	Biopsy	0	U	0
70	50	Female	Total resection	0	U	0,4
71	61	Male	Total resection	1	U	0
72	56	Male	Biopsy	0	U	0
73	73	Male	Total resection	2	U	0
74	49	Male	Total resection	0	U	0
75	63	Female	Partial resection	1	U	53,1
76	60	Male	Partial resection	0	U	81
77	71	Male	Total resection	0	U	0
78	65	Male	Total resection	2	U	99,9
79	64	Male	Total resection	0	U	0
80	40	Female	Partial resection	0	U	0
81	79	Male	Total resection	1	U	0
82	44	Female	Total resection	2	M	100
83	51	Male	Total resection	1	M	48,6
84	62	Male	Total resection	0	U	0
85	62	Male	Total resection	0	U	0
86	62	Female	Partial resection	0	U	0
87	71	Female	Biopsy	1	U	0
88	66	Male	Total resection	1	U	0
89	57	Male	Biopsy	0	U	35
90	69	Male	Partial resection	1	M	94,4
91	67	Female	Biopsy	1	U	6,9
92	59	Male	Partial resection	0	U	0
93	57	Female	Total resection	2	U	0
94	52	Male	Partial resection	0	U	0
95	50	Male	Partial resection	1	M	87,8
96	60	Female	Partial resection	2	M	91,3
97	58	Male	Biopsy	2	U	0
98	74	Female	Partial resection	1	M	47,9
99	25	Female	Biopsy	3	U	0
100	57	Female	Total resection	1	U	100

Age (years), M (methylated MGMT), U (unmethylated MGMT)

**Fig. 3:**
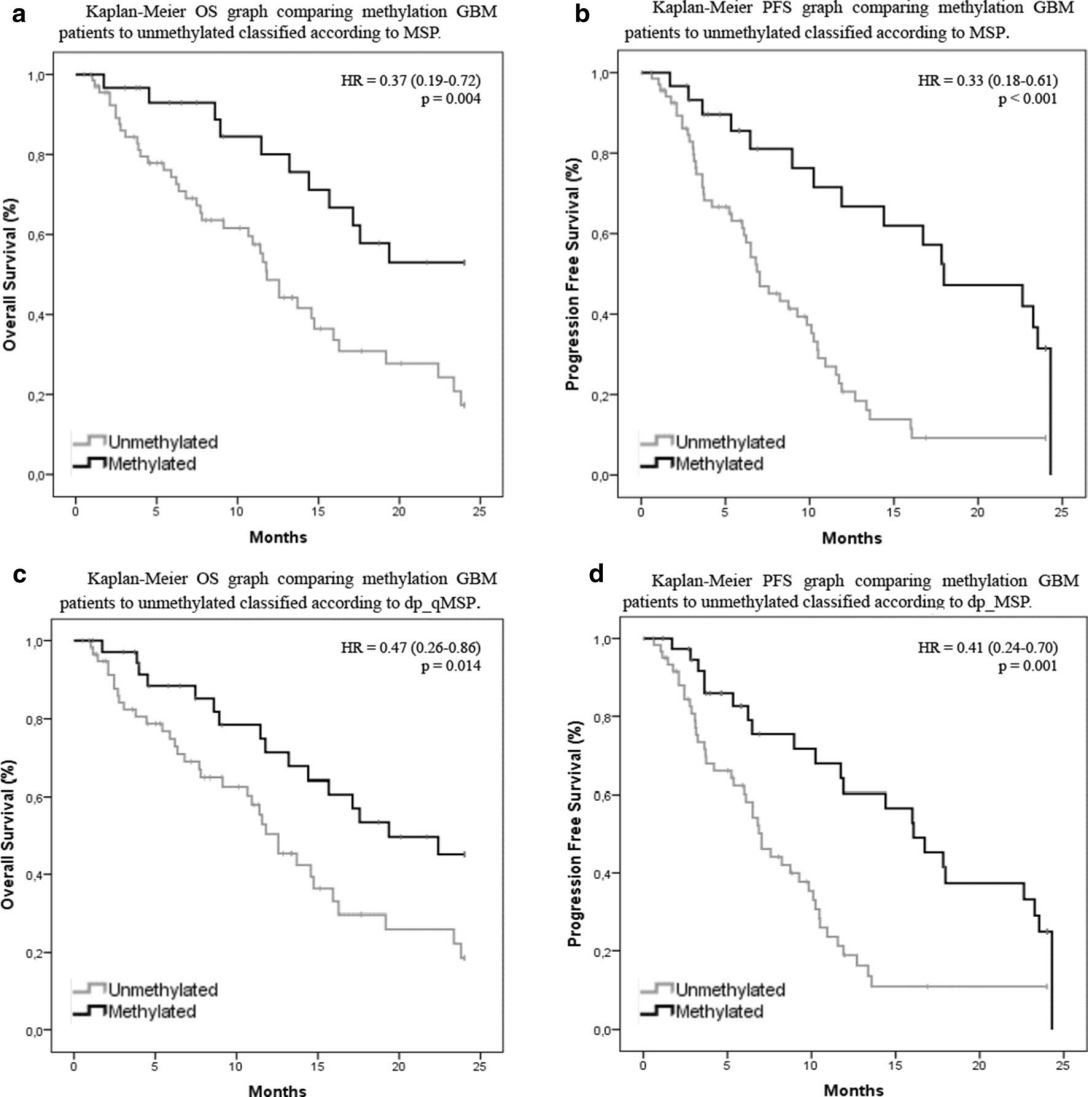
Survival analysis of GBM patients. **a** Kaplan–Meier OS graph comparing methylation GBM patients to unmethylated classified according to MSP. **b** Kaplan–Meier PFS graph comparing methylation GBM patients to unmethylated classified according to MSP. **c** Kaplan–Meier OS graph comparing methylation GBM patients to unmethylated classified according to dp_qMSP. **d** Kaplan–Meier PFS graph comparing methylation GBM patients to unmethylated classified according to MSP

### Examination dp_qMSP and MSP methods for survival analysis

The multivariable COX regression survival analysis identified significant differences for the variables MSP and dp_qMSP for both PFS (*p* = 0.001 and *p* = 0.004) and overall survival (*p* = 0.008 and *p* = 0.036) respectively; while no significant differences were found for the clinical variables (type of surgery, age, sex and ECOG). Therefore, we proceeded to study these variables using a univariate model. The median of OS measured by MSP in the group of patients with unmethylated *MGMT* promoter in our cohort was 11.8 months (95% CI 10.4–13.2) while the median of OS was not reached in the methylated group (Fig. 3a). We observed significant differences between unmethylated and methylated groups in terms of OS (*p* = 0.004, HR = 0.37, 95% CI 0.19–0.72). The rate of OS at two years was only 17% in the unmethylated group compared with the 53% observed in the methylated group. The median PFS was 7.0 months (95% CI 5.3–8.8) in the unmethylated MSP group and 18.0 months (95% CI 9.8–26.1) in the methylated MSP group (Fig. 3b). We also observed significant differences in terms of PFS regarding the methylation status between groups (p < 0.001, HR = 0.33, 95% CI 0.18–0.61). The rate of PFS at two years was 9.2% in the unmethylated group compared to the 31.5% observed in the methylated group. When using *MGMT* methylation data obtained by dp_qMSP, the median OS in the unmethylated group was 12.6 months (95% CI 10.0–15.1) while this median was not reached in the methylated group (Fig. 3c). Consistent with the results obtained by MSP, there were significant differences between unmethylated and methylated groups in terms of OS (*p* = 0.014, HR = 0.47, 95% CI 0.26–0.86) and PFS (*p* = 0.001, HR = 0.41, 95% CI 0.24–0.70). The rate of OS at two years was 19% in the unmethylated group compared to 45% in the methylated group. The median PFS was 7.0 months (95% CI 5.6–8.4) in the unmethylated dp_qMSP group and 16.0 months (95% CI 11.8–20.3) in the methylated dp_qMSP group (Fig. 3d). While the rate of PFS at two years was 10.8% in the unmethylated group compared to the 24.9% observed in the methylated group.

### Comparison between dp_qMSP and MSP methods for progression evaluation according to ROC (t)

**Fig. 4:**
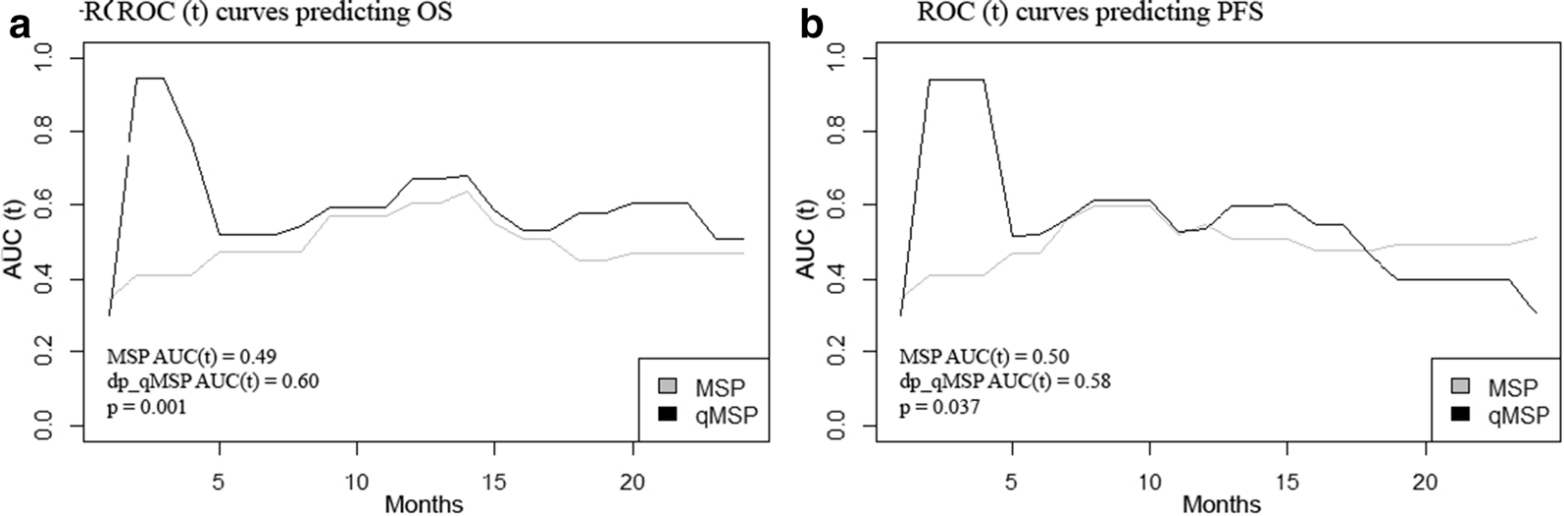
ROC (t) curves predicting OS and PFS. **a** AUC(t) for OS is higher for dp_qMSP than MSP (*p* = 0. 001). **b** AUC(t) for PFS is higher for dp_qMSP than MSP (*p* = 0.037)

We performed ROC(t) curves to compare both MSP and dp_qMSP for predicting PFS and OS in our cohort of GBM patients. The time-dependent area under the curve or AUC(t) for OS was 0.49 when we analyzed the patients with the MSP method and 0.60 in dp_qMSP assay (*p* = 0. 001). The AUC(t) for PFS was 0.50 when we analyzed the patients with MSP method and 0.58 in dp_qMSP assay (*p* = 0.037) (Fig. 4).

## Discussion

Food and Drug Administration and National Institutes of Health Biomarker Working Group define validation as a process to establish that the performance of a test is acceptable for its intended purpose [26]. In order to establish if the dp_qMSP test is suitable for *MGMT* methylation analysis, we performed comparison-of-methods studies between both dp_qMSP and MSP processes.

Firstly, we carried out a ROC study establishing the best cutoff methylation point at 3.75% and finding an excellent model (AUC = 0.962). The sensitivity obtained was 100% and we detected eight additional positive samples that were not identified by MSP. Being strict, we considered them as false-positive, decreasing our specificity to 88.6%; although they could certainly be due to an increased sensitivity of our methodology compared to the MSP. In fact, the clinical response in terms of survival in these patients corresponds to the mean of patients harboring a methylated promoter or to the overall mean of survival in this pathology, but not lower; except in two cases that did not could undergo resection surgery and therefore, a worse prognosis was expected, as described in the literature [27–29].

It has been reported that different methodologies could give rise to different results. When Quillen et al.compared five methods to analyze *MGMT* methylation, found in their study various discrepancies between the different assays used. Methylation-sensitive HRM and MethyLight obtained a weaker predictive value, whereas pyrosequencing was the best among the 5 techniques tested. In addition, Quillen’s study confirmed effectiveness as prognostic value of *MGMT* promoter methylation assessed by MS-PCR [14]. The subsequent study of Yoshioka et al.confirmed these good results obtained by the MS-PCR [16]. Dp_qMSP is based on MS-PCR, but it is improved by combining the PCR chemistry with amplicon detection by double fluorescence probes with a MGB, which stabilizes the double-stranded probe template structure resulting in improved allele specificity [30]. Moreover, qPCR can exclude ambiguity of interpretation which may cause bias in conventional PCR and it presents an easier workflow [31]. Thus, if we also take into account that qPCR presents higher sensitivity than the SYBERgreen-stained and gel-based detection under ultraviolet light we should not consider the new methylated samples identified by dp_qMSP to be false positives when using dp_qMSP, but rather that they are false negatives when using MSP. Furthermore, these results are supported by the parallel analytical validation of five of these samples performed in the MD Anderson Cancer center by using a modified methodology. Another possible cause that would explain this discrepancy is that the CpG 82 and/or 83 positions where our hydrolysis probe directed, could be methylated. Methylation of these positions would result in a positive result for dp_qMSP but could result in a negative result for MSP since in this methodology, these CpG positions are not considered.

With this in mind, we carried out further studies in order to decide the adequacy of both assays in terms of OS and PFS. The Kaplan–Meier analysis showed that patients with *MGMT* promoter methylation resulted in significantly longer PFS and OS than unmethylated patients, the same results as previously reported independently of using MSP or dp_qMSP [7, 14]. Therefore, both techniques allow the classification of patients as responders or non-responders in terms of *MGMT* methylation.

However, the question to be addressed is how well does the *MGMT* methylation biomarker evaluated by dp_qMSP distinguish between patients who respond to treatment and patients who do not at a given follow-up time. Cancer outcomes are very time-dependent and ROC curves that vary as a function of time may be more useful that the Kaplan–Meier analysis [32]. Therefore, ROC (t) has been used to compare the MSP and dp_qMSP and to establish the one that best fits the survival data. The AUC (t) for OS and PFS obtained was higher when we used the dp_qMSP method (0.49 versus 0.60 in OS and 0.50 versus 0.58 in PFS). We found significant differences between MSP and dp_qMSP, suggesting that the dp_qMSP assay might be more effective at detecting the *MGMT* methylation biomarker than the classic MSP assay.

**Fig. 5:**
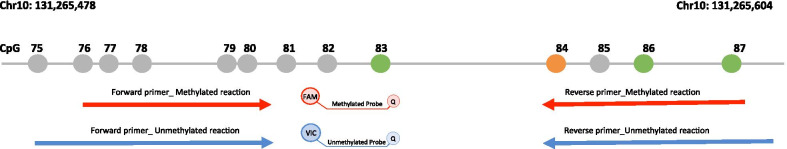
DNA and CpG island locations throughout the *MGMT* gene region (NM_002412; Chr10: 131,265,478—131,265,604). The CpG are represented as circles. The green circles symbolize the critical CpGs described by Malley et al. The orange circle symbolizes the cg12981137 described by Bady et al. The red and blue arrows represent M and U primers respectively. Between the primers, the hydrolysis probes labeled with two different reporter fluorochromes specific for recognizing methylated or Unmethylated DNA

In addition to the aforementioned advantages of using the dp_qMSP method, we may have obtained better results in AUC (t) particularly within the first 5 months after diagnosis in dp_qMSP because we are investigating the most important positions that have been shown to have a major impact on *MGMT* expression. Malley et al.described the methylation status of CpGs 83, 86, 87 and 89 as critical for transcriptional regulation, being the CpG 83 in our hydrolysis probe and CpG 86 and 87 in reverse primer. In addition, we have in our reverse primer the cg12981137, described by Bady et al.as one of the two more essential [10, 11] (Fig. 5)*.*

Due to the absence of adequately standardized techniques, international harmonization of the *MGMT* biomarker is still an unmet clinical need. A main difficulty has been the lack of a gold standard for *MGMT* methylation detection independently of the technology used. This is in part due to the different CpGs interrogated within the same technology, as there is still no consensus on how many CpG sites should be explored. For example, for pyrosequencing the cut-off values range from 2.7 to 35%, and the positions analyzed from four to more than 60 [15]. Several studies reported pyrosequencing as the method of choice for *MGMT* promoter methylation analysis in routine clinical practice [14, 33, 34] but, the current limitation of pyrosequencing is the absence of a consensus regarding an established cutoff for binary classification and concerning which are the most relevant CpG sites to analyze for clinical practice (as there are several pyrosequencing protocols that differ in regards to the number and position of the studied CpG sites) [15, 35–37]. The cutoff in pyrosequencing is calculated with the average of the different CpG positions analyzed by this technique and in some cases it gives rise to an indeterminate value called “gray zone” that is not capable of dichotomizing the cases, and there is no consensus on which of them most highly correlated with prognosis. In fact a recent article proposes to change this pyrosequencing calculation for a new analysis that could accurately predict the prognosis of patients in this "gray zone" [38] however, these data have not yet been validated. For all these reasons, *MGMT* methylation status has sometimes suffered from inconsistent results in the same tumor with different methods, mainly due to the lack of methodological standardization.

Undoubtedly and regardless of the methodology used, the settings for selecting the cutoff value, need to be identified with specific controls, allowing the results from each laboratory to be adapted according to the methodology used for DNA extraction, DNA bisulfite modification and the subsequent amplification method selected. We used MSP as a reference because it was the first method described and has been repeatedly shown to be of predictive value in randomized clinical trials [39–42]. However, MSP is a not an automatized method, making it difficult to standardize, and results may be influenced by tumor heterogeneity and/or a subjective interpretation. One of the great advantages of dp_qMSP is that the amplification of both methylated and unmethylated reactions, are carried out in the same PCR mixture, obtaining an accurate approximation of the percentage of one versus the other methylation status in a single sample. Moreover, dp_qMSP interrogates the most relevant positions that have been described in the literature, CpG 83, CpGs 86, CpG 87 as the best targets for methylation testing and critical for transcription regulation with the highest impact on *MGMT* expression and CpG 84 [10], described as one of the two essential positions that exhibited the strongest association with overall survival [11]. Furthermore, this amplification, in our experience, is reproducible when using different real-time PCR equipment such as the HT7900 or Step-One plus, which together with the use of a mathematical algorithm, would allow standardization between laboratories, as has been reported in the MethyLight technology, used for other epigenetic markers. The main advantage of dp_qMSP in this regard is that, unlike MethyLight, both reactions are amplified in parallel in the same mixture, simplifying the interpolation on two different genes in separate reactions as it is done in Methylight. This would greatly favor its routine implementation in a clinical diagnostic laboratory, in addition to avoiding errors related to greater technical complexity.

## Conclusion

Our study presents the evaluation of two techniques used for *MGMT* methylation: a standard MSP and a dp_qMSP, developed in our laboratory. The dp_qMSP method used in this study was not only more sensitive but also more time-efficient for the detection of *MGMT* biomarker. The method is based on qPCR assay that is carried out using two fluorochrome-labeled probes to quantify the percentage of methylated molecules in the tumor sample. We concluded that this assay is highly sensitive and easy to use with a well-established cutoff point, making it reliable and suitable for clinical use. Nevertheless, more multicenter studies are needed to confirm these results.

## Materials and methods

### Patient samples

Formalin-fixed paraffin-embedded (FFPE) GBM samples from 100 patients prospectively collected at University hospital La Paz, who underwent surgery or biopsy at the La Paz University Hospital between May 2014 and March 2020 were included in this study. The percentage of tumor cells in the tissue was evaluated previously by an expert pathologist to ensure the quality of the tumor sample. Inclusion criteria encompassed patients with signed informed consent, 18 years of age or older, Eastern Cooperative Oncology Group (ECOG) performance status of 0 to 3, histologically confirmed as a IDH-wild type GBM and treated with temozolamide. All patients had a minimum follow-up of 3 months.

The demographic information of all the patients as well as type of surgery, treatment, progression-free survival (PFS) and overall survival (OS) were collected. This study was conducted under the approval of the ethics committee of the La Paz University Hospital with the ethics number of PI-2887 and in conformance with the principles of the Declaration of Helsinki. Written informed consent was obtained from all participants.

### Nucleic acid isolation

Formalin-fixed and paraffin-embedded (FFPE) tissue samples were deparaffinized using xylene. DNA from tissue samples and cells were extracted according to standard protocols using phenol–chloroform, chloroform and finally suspending in 20 μl of 1X TE [43].

### Bisulfite modification of DNA

1000 ng of DNA from FFPE tissue was denatured by NaOH (0.2 mol/L) for 10 min at 37 °C and then modified by hydroquinone and sodium bisulfite treatment at 50 °C for 17 h. Modified DNA was purified using the Wizard DNA Clean-Up system (Promega, Madison, WI). Modification was completed by NaOH (0.3 mol/L) treatment for five minutes at room temperature followed by precipitation with glycogen, 10 mol/L ammonium acetate, and ethanol precipitation. Bisulfite modification of DNA resulted in the conversion of unmethylated cytosines to uracil, whereas methylated cytosines were resistant to modification and remained as cytosine [44].

### Nested-PCR

We performed a nested PCR to improve sensitivity and specificity. The reaction mixture contained 1.1 μl of bisulfite-modified DNA, 3 μl buffer (Biotools Buffer 10X), 4 μl dNTPs (10 mM), 1 μl MgCl_2_ (50 nM), 2 μl primers (100 ng/µL) and 0.75 μl (Biotools DNA polymersa 1U/µl) enzyme in a total reaction volume of 20.5 μl. Nested PCR conditions were 94ºC for 5 min, followed by 40 cycles at 94 ºC for one min, 54 ºC for one min, 72 ºC for one min and 72ºC for eight min. The product of the reaction is a 274 bp amplicon. The sequences (5′-3′) used were as follows: forward primer (BS_F) GGATATGTTGGGATAGTT and reverse primer (BS_R) CCGAAAAAAAACTAAACAACACCT. Amplicons resulting from this PCR reaction were used as template for MSP and dp_qMSP.

### Methylation-specific PCR (MSP)

The *MGMT* methylation status of patients’ samples was determined in the clinical routine by MSP. Consequently, amplicons resulting from nested-PCR were amplified using specific primers that discriminate methylated (M) and unmethylated (U) DNA previously described by Esteller et al.[45].

Primer sequences for M and U PCR reactions are as follows: for the methylated sequence MGMT_MF, TTTCGACGTTCTAGGTTTTCGC; MGMT_MR: GCACTCTTCCGAAAACGAAACG and for the unmethylated sequence MGMT_UF, TTTGTGTTTTGATGTTTGTAGGTTTTTGT; MGMT_UR, AACTCCACACTCTTCCAAAAACAAAACA. Primers for U reactions were analogous to M reactions, except CG was replaced with TG in the forward primer or CA in reverse primers. In addition, the U primers have additional base pairs, to be able to distinguish both reactions in an acrylamide gel. Primers were designed for the detection of an 81 bp fragment obtained from the methylated reaction and a 93 bp fragment from the unmethylated reaction. Peripheral blood mononuclear cell (PBMC) was used as unmethylated control and PBMC methylated in vitro with the enzyme CpG-Methyltransferase (M.SssI) was used as the methylated control.

The MSP reaction was performed in a final volume of 25 µl containing 1.1 µl of amplicons resulting from nested-PCR, 4.4 μl dNTPs (10 mM), 0.4 μl of each primers (100 ng/µL), 0.5 μl DNA polymerase (Biotools DNA polymersa 1U/µl) and 11.3 μl H2O. The MSP reaction was carried out with the following settings: for M reaction, 37 cycles of 95 °C for one min, 68 °C for one min, and 72 °C for 50 s; for U reaction, 35 cycles of 95 °C for one min, 60ºC for one min, and 72 °C for 50 s. The MSP products were electrophoresed on a 6% non-denaturing acrylamide gels with appropriate size markers and the presence or absence of a PCR product analyzed under ultraviolet light. A sample is considered methylated when amplification of a band is observed in both reactions, methylated and unmethylated (in the sample there will always be DNA of non-tumor origin that will be amplified as unmethylated). A sample is considered unmethylated when only a band amplification is observed in the reaction of the modified and unmethylated DNA specific primers.

### Quantitative methylation-specific PCR (dp_qMSP)

MGMT-dp_qMSP is a customized quantitative real-time PCR (qPCR) that specifically detects methylated and unmethylated bisulfite-modified DNA molecules from the same chromosomic location, specifically at the MGMT promoter area.

The dp_qMSP assay was carried out using ABI Prism 7900HT (Applied Biosystems, Darmstadt, Germany). We have used the same primers as those used for the MSP. In addition, we designed two hydrolysis probes, specifically for methylated or unmethylated DNA molecules. The methylated MGMT hydrolysis probe was labelled with a FAM fluorochrome at the 5′ end (FAM-CAAATCGCAAACGATA-MGB-NFQ) and the unmethylated MGMT hydrolysis probe was labelled with a VIC fluorochrome at the 5′ end (VIC-CAAATCACAAACAATA-MGB-NFQ). Both probes have a non-fluorescentquencher (NFQ) with a minor groove binder (MGB) at the 3′ end. Hydrolysis probes for M and U reactions were identical, except at CpG sites, which were unique for recognizing M or U positions.

The amplification mixture consisted of 9.5 μl of DNA Master Mix (QuantiTect Multiplex PCR Master Mix, QIAGEN), 8.4 μl of H_2_O, 0.125 μl each primer (280 ng/µL), 0.06 μl each probe (100 µM), and 3.5 μl of template DNA in a final volume of 20 μl. PBMC was used as unmethylated control and PBMC in vitro methylated are used as the methylated control. qPCR reaction involved an initial denaturation at 95 ºC for 15 min, followed by 40 cycles of 94 ºC for one min and 60 ºC for one min. Data acquisition and analysis was performed on the RQ Manager 1.2.1 software. The percentage of methylation was carried out using the formula previously described = 100/(1 + (2^(Cq_methylated-Cq_unmethylated)))[25].

### Statistical analysis

The association between methylation and clinicopathological status (qualitative variables) were analyzed using the chi-square test (type of surgery, ECOG) or Fisher's exact test (gender). For the comparison between qualitative methylation and age (quantitative data), the t-student test for independent data was used. Receiver Operating Characteristics (ROC) analysis was performed to determine the optimal cutoff for dp_qMSP. Samples were categorized as methylated or unmethylated based on the cutoff determined through ROC curve analysis. The sensitivity and specificity were obtained using MSP as the reference. In addition, time-dependent ROC curves or ROC(t) were used for evaluating and comparing the prognostic value of the *MGMT* methylation marker between MSP and dp_qMSP[32].

The survival analysis were carried out using the Kaplan–Meier analysis and the Cox regression. In order to compare the survival functions by groups, log-rank tests were performed. Differences were considered statistically significant at *p* ≤ 0.05. Confidence intervals (CIs) were made using a 95% confidence level. Statistical analysis was conducted by a biostatistical expert using the SAS 9.3 program (SAS Institute, Cary, NC, USA) and R version 4.0.0.

## Supplementary Information

**Additional file 1: Supplementary Figure 1**. Uncropped gel from Fig. 2.

**Additional file 2: Supplementary Figure 2**. Examples of MSP and dp_qMSP amplifications from the same samples. **A** MSP samples are shown in duplicate U: Unmethylated reaction, M: methylated reaction, C- : Unmethylated control sample and C+: IVD control sample. The water is tested for contamination. **B** dp_qMSP amplifications showing the FAM and VIC probes for each sample tested together with the positive and negative controls (only amplify the FAM and VIC probes respectively, there is no amplification when the VIC probe is used with the positive control and not the FAM probe when the negative control is tested). For sample number 100 there is only amplification with the FAM probe (100% methylation). **C** Reflects the percentage of methylation in each case after application of the formula.

**Additional file 3: Supplementary Figure 3**. Agarose gel with the MGMT promoter amplification of five samples with discrepancies between MSP and dp_qMSP. It has been performed in a collaborative center (MD Anderson Madrid) with an alternative methodology using EpiTec for DNA modification and using the next primers and conditions for PCR amplification: MGMT-M-F TTTCGACGTTCGTAGGTTTTCGC and MGMT-M-R GCACTCTTCCGAAAACGAAAC MGMT-U-F TTTGTGTTTTGATGTTTGTAGGTTTTTGT and MGMT- U-R AACTCCACACTCTTCCAAAAACAAAACA For both reactions the PCR settings are 58°C and 35 cycles. The arrows indicate a slightly amplification at the methylation reaction in samples 1, 78 and 100. For none of those patients these results were considered positive for clinical diagnosis.

## Data Availability

The dataset supporting the conclusions of this article are included within the article and its additional file.

## Abbreviations

MGMT: Methylguanine-DNA methyltransferase
GBM: Glioblastoma
DMR2: Differentially methylated region
OS: Overall survival
MSP: Methylation-specific PCR
MS-MLPA: Multiplex ligation-dependent probe amplification
HRM: High-resolution melting
COBRA: Combined bisulfite restriction analysis
qMSP: Quantitative methylation specific PCR
FFPE: Formalin-fixed paraffin-embedded
ECOG: Eastern Cooperative Oncology Group
PFS: Progression-free survival
ROC: Receiver Operating Characteristics
M: Methylated
U: Unmethylated
qPCR: Quantitative real-time PCR
NFQ: Non-fluorescent quencher
MGB: Minor groove binder
CI: Confidence interval
AUC: Area under the curve
