# Supplementary figures and images for: Clinical validation of a novel quantitative assay for the detection of MGMT methylation in glioblastoma patients
Source: Clin Epigenetics. 2021 Mar 9;13:52. doi: 10.1186/s13148-021-01044-2 (PMC7941980; doi:10.1186/s13148-021-01044-2)

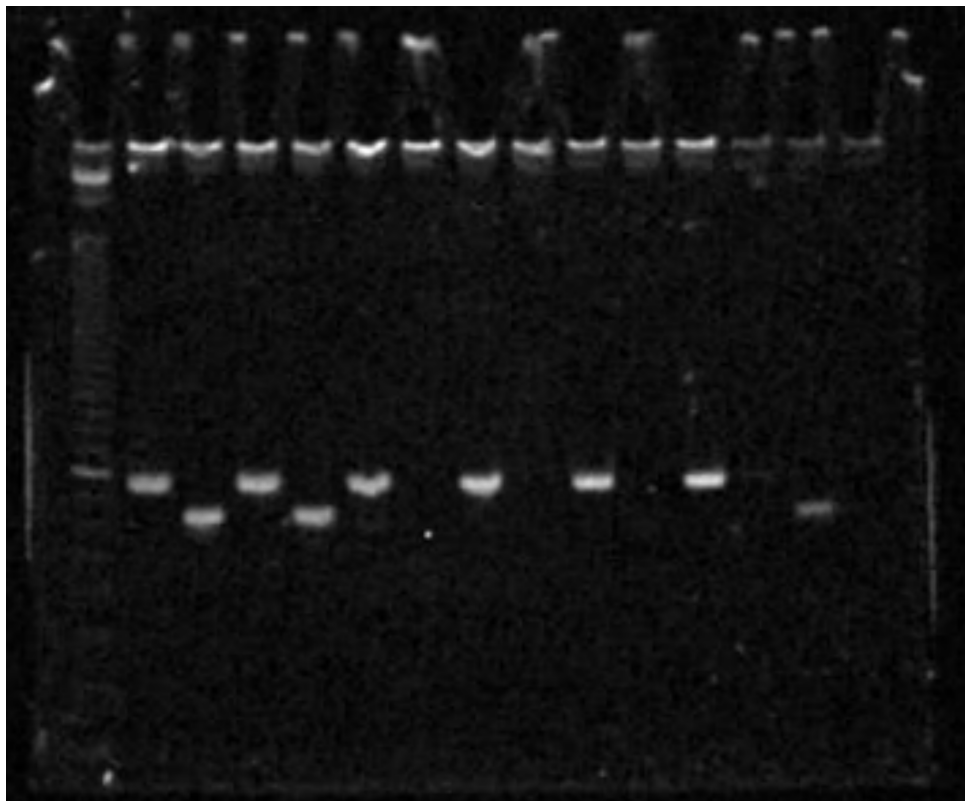

Supplementary Figure 1: Uncropped gel from Figure 2

Supplement: Supplementary file 1 — Additional file 1: Supplementary Figure 1. Uncropped gel from Fig. 2. [file 13148_2021_1044_MOESM1_ESM.pdf]
