# Supplementary material for: Clinical validation of a novel quantitative assay for the detection of MGMT methylation in glioblastoma patients
Source: Clin Epigenetics. 2021 Mar 9;13:52. doi: 10.1186/s13148-021-01044-2 (PMC7941980; doi:10.1186/s13148-021-01044-2)
Supplement: Supplementary file 2 — Additional file 2: Supplementary Figure 2. Examples of MSP and dp_qMSP amplifications from the same samples. A MSP samples are shown in duplicate U: Unmethylated reaction, M: methylated reaction, C- : Unmethylated control sample and C+: IVD control sample. The water is tested for contamination. B dp_qMSP amplifications showing the FAM and VIC probes for each sample tested together with the positive and negative controls (only amplify the FAM and VIC probes respectively, there is no amplification when the VIC probe is used with the positive control and not the FAM probe when the negative control is tested). For sample number 100 there is only amplification with the FAM probe (100% methylation). C Reflects the percentage of methylation in each case after application of the formula. [file 13148_2021_1044_MOESM2_ESM.pdf]

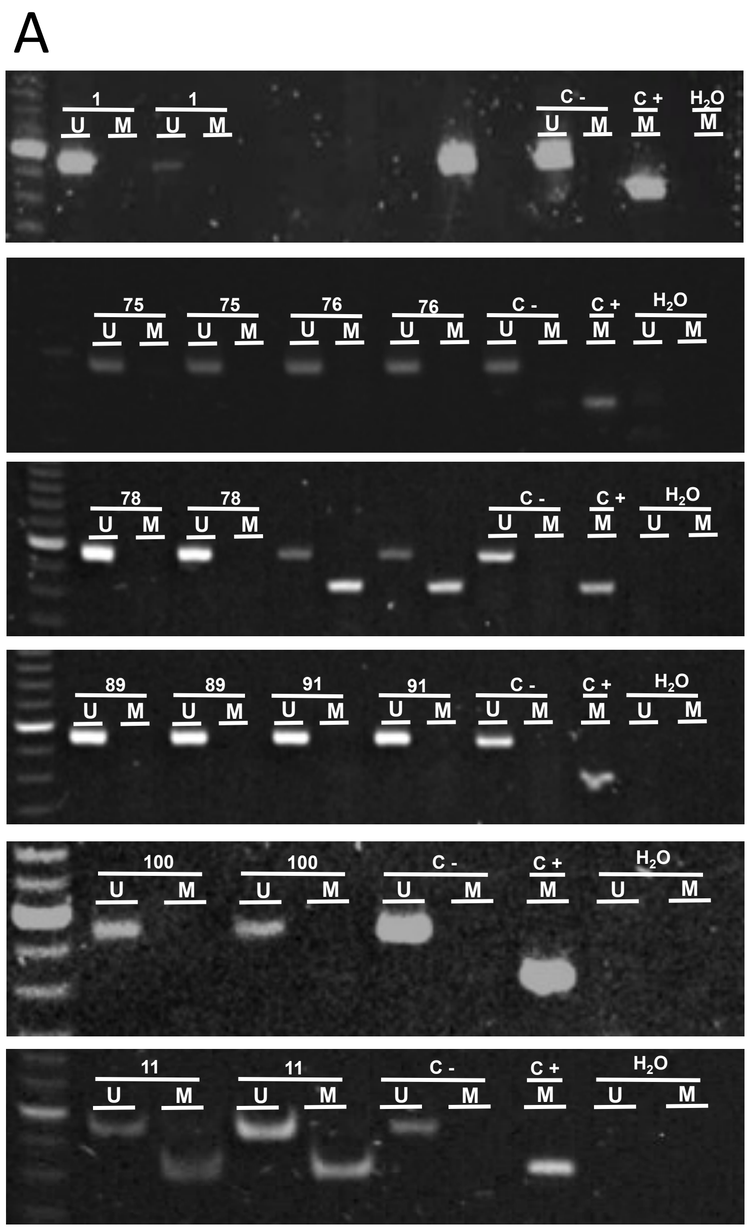

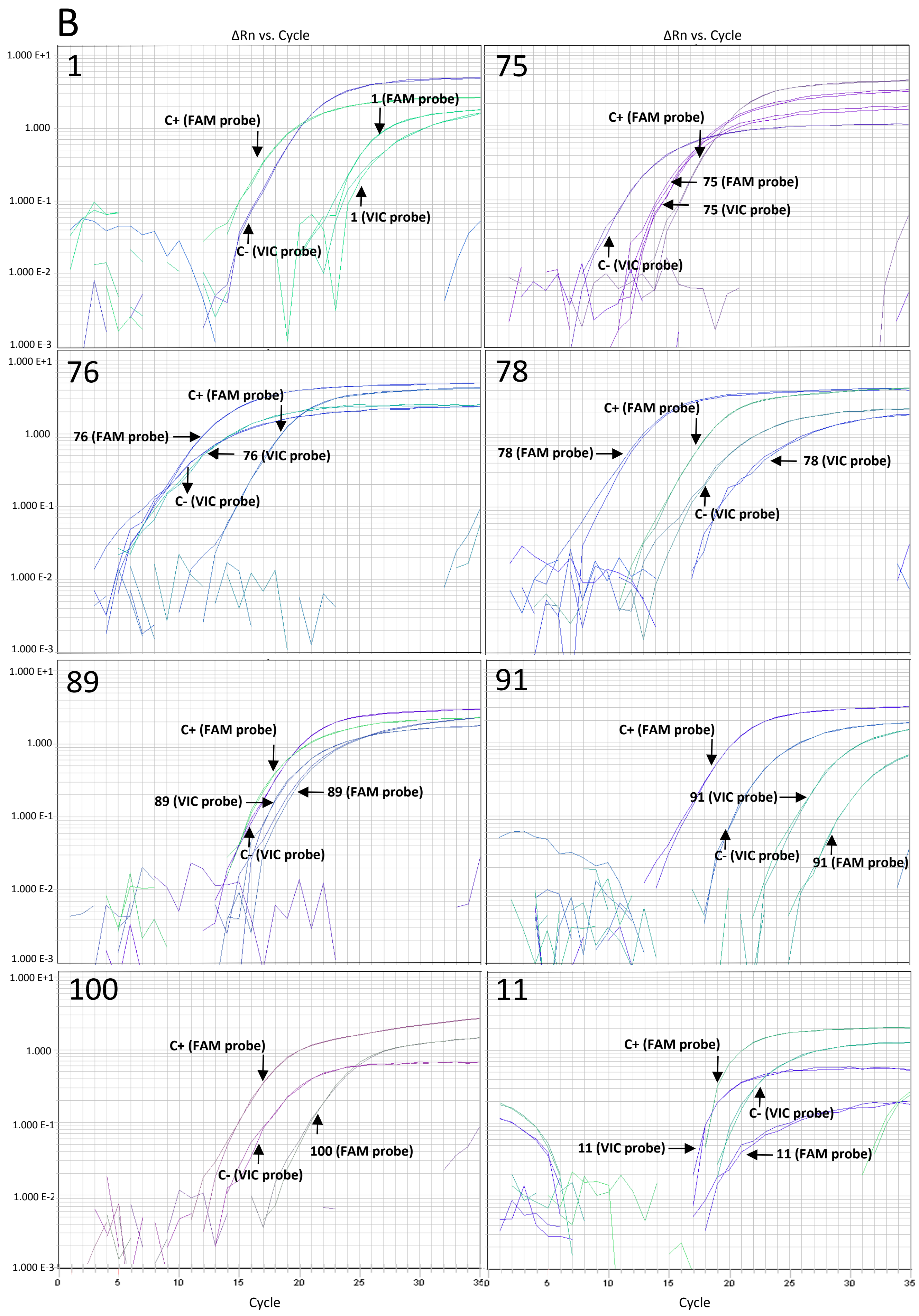

C

ID	% Meth	ct FAM	ct VIC
1	66,7	23,2	24,2
75	53,1	16,2	16,38
76	81,1	11,2	13,3
78	99,9	10,4	21,7
89	35	19,05	18,16
91	6,9	29,65	25,9
100	100	22	No Amplification
11	5,9	21,5	17,5

Supplementary Figure 2. Examples of MSP and dp_qMSP amplifications from the same samples. **A.** MSP samples are shown in duplicate U: Unmethylated reaction, M: methylated reaction, C- : Unmethylated control sample and C+: IVD control sample. The water is tested for contamination. **B.** dp_qMSP amplifications showing the FAM and VIC probes for each sample tested together with the positive and negative controls (only amplify the FAM and VIC probes respectively, there is no amplification when the VIC probe is used with the positive control and not the FAM probe when the negative control is tested). For sample number 100 there is only amplification with the FAM probe (100% methylation). **C.** Reflects the percentage of methylation in each case after application of the formula.
