# Supplementary material for: Clinical validation of a novel quantitative assay for the detection of MGMT methylation in glioblastoma patients
Source: Clin Epigenetics. 2021 Mar 9;13:52. doi: 10.1186/s13148-021-01044-2 (PMC7941980; doi:10.1186/s13148-021-01044-2)
Supplement: Supplementary file 3 — Additional file 3: Supplementary Figure 3. Agarose gel with the MGMT promoter amplification of five samples with discrepancies between MSP and dp_qMSP. It has been performed in a collaborative center (MD Anderson Madrid) with an alternative methodology using EpiTec for DNA modification and using the next primers and conditions for PCR amplification: MGMT-M-F TTTCGACGTTCGTAGGTTTTCGC and MGMT-M-R GCACTCTTCCGAAAACGAAAC MGMT-U-F TTTGTGTTTTGATGTTTGTAGGTTTTTGT and MGMT- U-R AACTCCACACTCTTCCAAAAACAAAACA For both reactions the PCR settings are 58°C and 35 cycles. The arrows indicate a slightly amplification at the methylation reaction in samples 1, 78 and 100. For none of those patients these results were considered positive for clinical diagnosis. [file 13148_2021_1044_MOESM3_ESM.pdf]

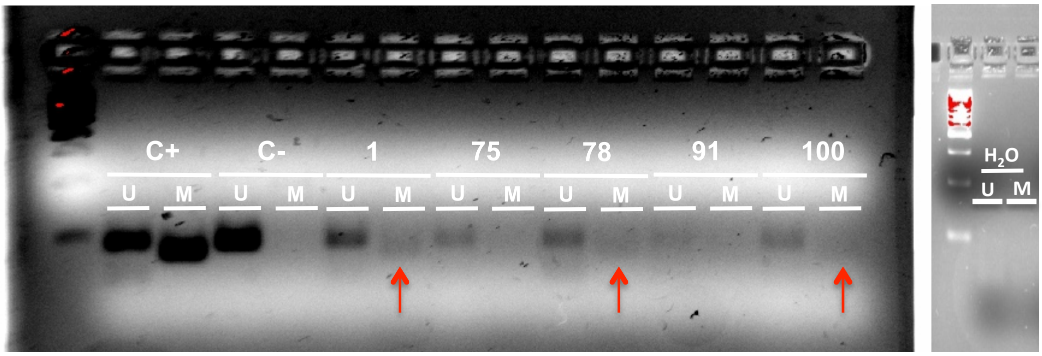

Supplementary Figure 3. Agarose gel with the MGMT promoter amplification of five samples with discrepancies between MSP and dp_qMSP. It has been performed in a collaborative center (MD Anderson Madrid) with an alternative methodology using EpiTec for DNA modification and using the next primers and conditions for PCR amplification:

MGMT-M-F TTTCGACGTTCGTAGGTTTTCGC and MGMT-M-R GCACTCTTCCGAAAACGAAAC
MGMT-U-F TTTGTGTTTTGATGTTTGTAGGTTTTTGT and MGMT- U-R AACTCCACACTCTTCCAAAAACAAAACA

For both reactions the PCR settings are 58°C and 35 cycles.

The arrows indicate a slightly amplification at the methylation reaction in samples 1, 78 and 100. For none of those patients these results were considered positive for clinical diagnosis
